# Investigation about the Occurrence of Transmission Cycles of Arbovirus in the Tropical Forest, Amazon Region

**DOI:** 10.3390/v11090774

**Published:** 2019-08-22

**Authors:** Pedro A. Araújo, Maria O. Freitas, Jannifer Oliveira Chiang, Franko Arruda Silva, Liliane Leal Chagas, Samir Mansour Casseb, Sandro Patroca Silva, Joaquim Pinto Nunes-Neto, José Wilson Rosa-Júnior, Bruna Sena Nascimento, Leonardo Almeida Hernández, Thito Bezerra Paz, Landeson L. Barros, Eliana P. Silva, Raimunda S. Azevedo, Lívia C. Martins, Milene S. Ferreira, Pedro F. Vasconcelos

**Affiliations:** 1 Institute of Biological Sciences, Federal University of Pará, Belém, PA 66075-110, Brazil; 2 Department of Arbovirology and Hemorrhagic Fevers, Evandro Chagas Institute, Ananindeua, PA 67030-000, Brazil

**Keywords:** arbovirus, biodiversity, Amazon, Caixuanã, Pará, Brazil

## Abstract

Because of its ecological characteristics, the Caxiuanã National Forest (FLONA) is a potential area of arbovirus circulation. The present study aimed to investigate the occurrence of arbovirus transmission cycles at FLONA de Caxiuanã. Five field trips were performed to capture mosquitoes and sylvatic vertebrates. For these vertebrates, we attempted viral isolation by cell monolayer inoculation from blood, and hemagglutination inhibition and further seroneutralization assays from sera. For mosquitoes, we performed tests of viral genome detection. A total of 338 vertebrates were captured, and the greatest representative was birds (251/74.26%). A total of 16,725 mosquitoes were captured, distributed among 56 species. There were no viruses isolated by newborn mouse inoculation. Among birds, antibodies against *Ilheus virus* were the most prevalent. *Catu virus*, *Caraparu virus*, and *Mucambo virus* were the most prevalent among mammals and reptiles. Fragments of *Mucambo virus*, *Ilheus virus*, *Bussuquara virus*, and *Rocio virus* genome were detected in a pool of mosquito samples. These results of the study suggest the occurrence of arbovirus transmission cycles in the FLONA of Caxiuanã. The proximity of human populations with elements, involved in transmission cycles, makes surveillance necessary in this population to avoid dispersion of arboviruses to naïve locations.

## 1. Introduction

Arboviruses are an extensive viral group that shares antigenic similarities and can be maintained in nature through transmission by infected hematophagous arthropods [1,2,3]. Transmission cycles of arboviruses usually occur in sylvatic environments and require the involvement of vertebrate hosts (in general, birds and small mammals) and arthropods vectors [3,4]. Humans often participate as accidental hosts and may become infected through biting by infected arthropods when they enter into ecosystems where there is viral circulation [5,6].

In Brazil, the endemicity of arboviruses is concentrated mostly in the Atlantic and Amazon forest biomes [7]. The Amazon region is considered an area of high endemicity for several arboviruses due to its great diversity of animals hosts and vectors, which makes the Amazon Forest one of the biggest reservoirs of arboviruses in the world [8].

Situated inside the Amazon Forest, the Caxiuanã National Forest (FLONA) has emerged as a location for possible circulation of arboviruses due to ecological features required for occurrence and maintenance of arbovirus transmission cycles.

To identify the species of vertebrates and mosquitoes that circulate in the FLONA of Caxiuanã and the interactions that occur between them is important for understanding how arboviruses established transmission cycles between these animals, how these cycles interfere in the dynamics of FLONA, as well as the impacts generated on human populations, due to proximity with cycle elements. In this sense, the present study aimed to investigate the occurrence of transmission cycles of arboviruses in the FLONA of Caxiuanã through attempts at viral isolation, detection of antibodies, and identification of fragments of arboviral genomes, from biological samples obtained from animals captured in the FLONA.

## 2. Materials and Methods

### 2.1. Study Areas

**Figure 1:**
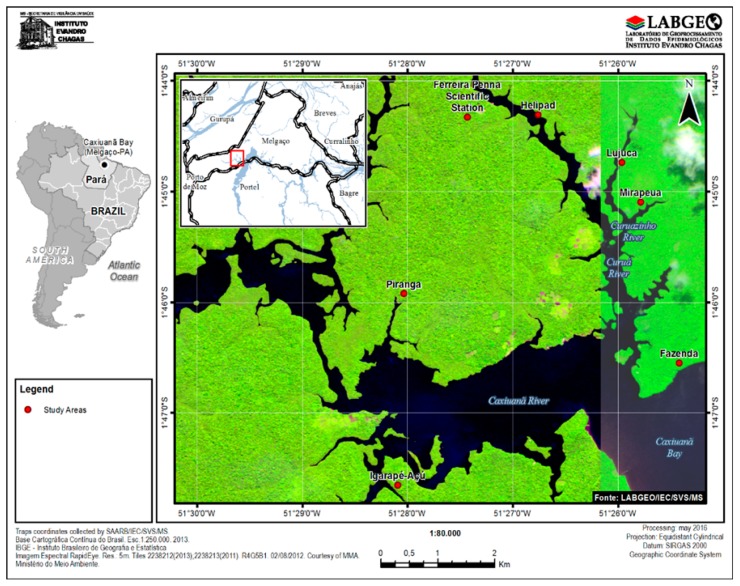
Cartographic representation of study areas at FLONA de Caxiuanã. Source: IEC. Green area: the continent; Blue area: the river.

This study was carried out on FLONA of Caxiuanã (S1°44’14.15” W051°27’19.53”), situated on Marajó’s archipelago, State of Pará, during the period November 2014 to March 2016. A total of five field trips were performed, resulting in 338 biological samples from sylvatic vertebrates, and 33,440 hematophagous arthropods were collected. The sites for the collection were Estação Científica Ferreira Pena, Heliporto, Lujuca, Mirapéua, Piranga, Fazenda, and Igarapé Açu (Figure 1).

### 2.2. Collection Methods

Hematophagous arthropods were captured on the ground and in the canopy of trees through two techniques: human attraction protected and enlightened at day, using hand nets (polyester net bag 30 cm in diameter, attached to a 30 cm aluminum handle, constructed by the research team) and an oral suction device, which stored captured specimens, and light attraction, using CDC light trap (John W. Hock Company, Gainesville, FL, USA) at night, from 18:00 to 6:00. After taxonomic identification, arthropods were organized in pools.

Birds were captured with nylon nets, and mammals and reptiles were caught using Shermann and Tomahawk traps at ground level. Animals were weighed, anesthetized, blood samples were taken, and then they were released. Anesthetic doses and blood volumes were calculated from weight measurements. Specimens found to be dead, sick, or that died during the collection procedures underwent necropsy *in loco*, and organs and sample fragments were stored in liquid nitrogen until further analysis.

### 2.3. Temperature and Humidity

To characterize climate period on field trips, daily measures of temperature and humidity were performed using GR-303 Thermo-Hygrometer (Shenzhen Graigar Technology Co., Ltd. Shenzhen, China) equipment.

### 2.4. Viral Isolation

For attempts at viral isolation, Vero A18 cells derived from African green monkey kidney epithelial tissue (*Chlorocebus sabaeus*) were employed [9]. Blood and viscera samples were inoculated in 199 maintenance medium containing tryptose, penicillin (100 UL/mL), streptomycin (100 UL/mL), and 2% fetal bovine serum (FBS) [10]. Cell cultures were observed for the following ten days to visualize cytopathic effects (ECP) on monolayers. The viral presence was confirmed by indirect immunofluorescence (IIF) assays, and tested against polyclonal (Group A, Group B, and Oropouche Virus) and monoclonal antibodies, following a previous protocol [11]. For samples with negative results for the presence of arbovirus, we performed three blind passages for the confirmation of these results.

### 2.5. Antibody Detection

Serological investigations were carried out using hemagglutination inhibition (HI) tests [12], adapted for microplates [13]. For such, the samples were initially treated with 100% acetone aiming to remove possible interferers of hemagglutinant activity, as well as induce the formation of precipitate containing antibodies. Subsequently, the samples were tested against a panel composed of 29 different arbovirus antigens, divided between *Alphavirus*, *Flavivirus*, *Orthobunyavirus*, and *Phlebovirus* genera (Table A1). Following contact of the precipitate with antigens, a detection system composed of goose red blood cells (RBC) was added. If antigen-antibody binding occurs, goose RBCs will remain free and will sediment in the well of the microplate. If this takes place, the sample is considered positive for the presence of antibodies against arbovirus. When antigen-antibody binding does not occur, the antigens bind to goose RBCs, promoting disruption of those cells. Then, the sample is considered negative.

Samples positive for HI testing were subjected to Neutralization Tests (NTs) in newborn mice [14]. Briefly, cerebral suspensions of mice infected with the investigated viruses were serially diluted from 10^−2^ to 10^−10^. The homologous sera of investigated viruses (positive control), the biological samples, and negative controls were diluted in 1:10 in FBS following incubation for 60 min at 37 °C. After incubation, 0.02 mL of the dilutions was inoculated via the intracerebral (IC) route in newborn mice, which were observed for the following 21 days. Lethal dose—LD_50/0.02 mL_ was calculated by the Reed and Muench method [15]. Samples were considered positive when they reached the log neutralization index (LNI) ≤1.7.

### 2.6. Viral Genome Detection

Viral RNA extraction of arthropods was performed on a Maxwell^®^16 System RNA (Promega, Madison, WI, USA) device, using Maxwell^®^ LEV 16 simplyRNA Tissue Kit (Promega, USA), following manufacturer’s instructions. For reverse transcription (RT) reactions, EasyScript^®^ First-Strand cDNA Synthesis SuperMix (TransGen Biotech Co. Ltd, Beijing, China) kit was used, including Random primer (N9) reagent, according to the manufacturer’s description. RT reactions were performed on a GeneAmp PCR System 9700 (Invitrogen, Carlsbad, CA, USA) thermocycler, with incubation cycles of 25 °C for 10 min, 42 °C for 30 min, and 85 °C for five seconds for enzymatic inactivation.

For Polymerase Chain Reactions (PCRs), biological samples were amplified by Platinum™ Taq DNA Polymerase (ThermoFisher Scientific, Waltham, MA, USA) enzyme in reactions volumes of 50 µL containing 10 µL of DNA previously reverse-transcribed template and 40 µL of reaction mix reagents, established by the manufacturer. Specific primers for viral genera were used for PCR amplification. The primers were chosen based on the literature and validated and tested in the laboratory using positive controls for several viruses of the viral genomes. The primers for *Alphavirus* (M2W(F) YAG AGC DTT TTC GCA YST RGC HW e cM3W(R) ACA TRA ANK GNG TNG TRT CRA ANC CDA YCC) amplify the 3’ region of the viruses of this genus. This region has a high similarity between these viruses and is considered a conserved area; this has already been described by Pffefer et al., 1998 [16]. The primers for *Flavivirus* (MA(F) CAT GAT GGG RAA RAG RGA RRA G e cFD2(R) GTG TCC CAG CCG GCG GTG TAC TAC GC) were described by Kuno et al. 1998 [17]. The amplified region lies between the 3’ and NS5 regions and is extremely well conversed since it is related to the viral polymerase. Therefore, it does not undergo significant alterations between the virus of the genre. The annealing temperature was set at 53 °C and 54 °C for *Alphavirus* and *Flavivirus*, respectively.

The amplified genome was processed on GeneAmp^®^ PCR System 9700 (ThermoFisher Scientific, USA) thermocycler and visualized on 1% agarose gels containing SYBR^®^ Safe DNA Gel Stain (ThermoFisher Scientific, USA) using UV L-Pix Transluminator (Loccus Biotecnologia, Cotia, Brazil).

### 2.7. Nucleotide Sequencing

The molecular characterization of obtained sequences was performed on MiniSeq (Illumina, Santiago, CA, USA) platform. The process commenced with second strand synthesis of ssRNA using a cDNA Synthesis System (Roche Diagnostics, Basel, Switzerland) kit, plus 400 µM of random Roche Primer. Reactions were then purified with an Agencourt AMPure XP Reagent (Beckman Coulter, Brea, CA, USA) kit.

A genomic cDNA library was prepared following Nextera XT DNA Library Preparation Kit instructions, on MiniSeq (Illumina) equipment. Viral genomes were assembled by de novo, using the IDBA-UD program [18]. Annotation of coding and non-coding DNA was performed by Geneious v.9.1.6 (Biomatters, Auckland, New Zealand) software. Multiple sequence alignments were performed using Mafft v.7 software [19].

### 2.8. Statistical Analysis

To evaluate correlations between the variables “climate period” and “number of captures”, Fisher’s exact test was applied. The significance level (*p*-value) was set as 5% (0.05), and confidence intervals were set at 95%.

### 2.9. Legal Aspects

The present study was approved by Instituto Chico Mendes de Conservação da Biodiversidade (ICMBio) through the Sistema de Informação de Biodiversidade (SISBio) under the protocol number 42761-1, emitted on 15th August 2014; and by the Committee of Ethics in the Use of Animals of Evandro Chagas Institute (CEUA/IEC), under the protocol number 027/2016, emitted on 11th August 2016.

## 3. Results

A total of 324 wild vertebrates were captured during the study period. Birds were the most prevalent vertebrate, comprising 251 specimens. The most abundant species was *Willisornis vidua* (“Rendadinho”), which represented 17.52% of the total (44 specimens). *Mammalia* class represented 65 specimens, and the *Oecomys* genus was the most frequent (*n* = 23; 35.38%). Only eight specimens of *Reptilia* class were captured, and *Ameiva fuscata* was the most prevalent, representing 50% (*n* = 4). A total of 16,725 specimens of mosquitoes were collected. Among the most prevalent: *Coquillettidia (Rhy.) venezuelensis* (*n* = 4574; 13.67%), *Culex (Mel.)* sp. (*n* = 4020; 12.02%), and *Culex (Mel.) portesi* (*n* = 2073; 6.19%).

Regarding seasonality, the greatest number of vertebrates was captured during the dry period and comprised 200 specimens (61.72%). Despite these results, statistical analysis of climate and number of animals collected showed no significant difference (Fisher’s exact test: *p* > 0.05). In contrast, the greatest number of arthropods collected was during the rainy season, comprising 11,383 (68.05%) specimens. Between *ecotopos* of arthropods collection – a canopy of trees and ground, the latter totaled 13,086 specimens (78.24%). Light traps recorded a large number of arthropods (*n* = 8897; 53.19%). The relation between climatic period and *ecotopo* of capture was statistically significant (Fisher’s exact test: *p* < 0.05). Fazenda was an area with a high frequency of collected animals, comprising 124 vertebrates (38.27%) and 6705 arthropods (40.08%). Lujuca was the second area with high numbers of captures, with a total of 67 vertebrates (19.82%) and 4078 mosquitoes (24.38%) collected.

**Figure 2:**
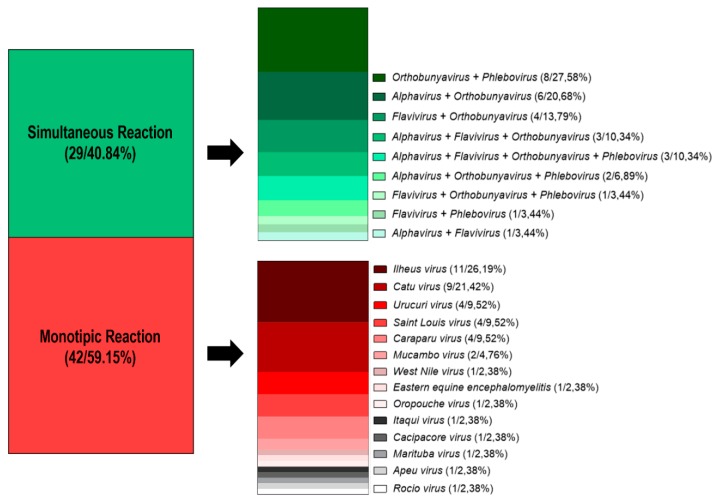
Quantitative and percentage of simultaneous and monotypic reactions detected in samples sera of wild vertebrates from FLONA of Caxiuanã between 2014 and 2016.

A total of 330 samples (blood and viscera samples) of vertebrates were collected during the study period. All were submitted to attempt viral isolation in Vero A18 cell cultures and showed a negative result. Due to the fragility of some vertebrates captured and an aim to avoid animal death, only 150 sera samples were collected and tested for HI, 71 (47.33%) of which were positive for the presence of antibodies against arboviruses. Among the positive samples, 42 (59.15%) showed monotypic reactions, that is, against only one viral genus. The greatest number of monotypic reactions occurred against the *Ilheus virus* (11/26.19%) and *Catu virus* (9/21.42%). In 29 (40.84%) samples, we observed simultaneous reactions against more than one viral genus. Such simultaneous reactions were more frequent among the *Orthobunyavirus* and *Phlebovirus* genera, followed by the *Alphavirus* and *Orthobunyavirus* genera (Figure 2).

Fourteen samples positive for HI were also tested for *Ilheus virus* (ILHV), *Mucambo virus* (MUCV), and *Oropouche virus* (OROV) by NT assays. In NT, a total of 14 sera samples were tested. Of these, six samples (85.71%) presented LNI greater than 1.7 and were considered positives for the presence of specific antibodies against MUCV (*n* = 5/35.71%) and OROV (*n* = 1/7.14%) (Table A2).

The total of 16,725 specimens of mosquitoes collected was grouped in 680 pools, according to species. These pools were tested for the presence of viral genomes of *Flavivirus* and *Alphavirus* genera. Four pools presented positive result, and the nucleotide sequencing detected the genome of *Mucambo virus, Ilheus virus, Bussuquara virus*, and *Rocio virus* (Table 1).

## 4. Discussion

In contrast to other studies that had success in isolating several viruses from the blood of birds collected in the field, in this work, it was not possible to detect any virus from such animals [20,21]. This can be explained by the short viremia and low serological titers observed in birds [22]. Moreover, there is great difficulty in determining viral load during viremia [23].

Many serological studies [7,24,25] involving free-living wild birds have reported a high prevalence of antibodies against the *Flavivirus* genus, corroborating our data. In contrast to birds, the wild mammals presented serological profile with more frequent detections of *Orthobunyavirus* genus, which is different to those observed in other investigations [7,25], which showed a high prevalence of *Flavivirus* genus antibodies.

For detection of antibodies involving *Orthobunyavirus* and *Flavivirus* genera, the greater number of reactions was against *Catu virus* and *Ilheus virus*, respectively. Concerning CATV, this fact may be attributed to the high circulation of vectors from such viruses. The species *Culex (Mel.) portesi* is considered the main vector of CATV, and several isolations of this arbovirus have been reported in Brazil [26] and other countries [27,28,29,30]. The high prevalence of antibodies against ILHV can be associated with the elevated circulation of mosquitoes from such *Culex sp* genus, which are normally associated with the transmission of several flaviviruses [31,32,33], mostly from the Japanese encephalitis serological complex [34], of which ILHV is part.

Neutralization tests indicated the presence of antibodies against MUCV and OROV in mammals and birds of *Xyphorhynchus* genus samples, respectively. Other studies have also demonstrated the presence of specific antibodies against OROV [35,36,37] in wild birds of *Xyphorhynchus* genus [38], and specific antibodies against MUCV [39] in rodents.

Data obtained in this work ratify other studies that sought to shed light on wild fauna serology. Our results demonstrated that rodents and birds might be evolved in viral transmission cycles and harbor MUCV and OROV, respectively [20,40,41].

A serological survey carried out in the human population, a resident in the FLONA, demonstrated that, just like the wild animals, these humans were likely to participate in transmission cycles of certain arboviruses. Indeed, IgM antibodies against ROCV and ILHV were detected, with a prevalence of 24% and 16%, respectively, indicating a recent or current infection. Furthermore, neutralizing antibodies against MUCV were detected, reaching 57% prevalence among the total number of sera tested [42].

The detection of BSQV, ROCV, and ILHV genome fragments in pools of *Cx. (Mel.) portesi*, and of MUCV in *Uranotaenia (Ura.) geometrica* showed the real necessity for more studies to shed light on the participation of such species in the arbovirus transmission cycles. Despite such evidence, the lack of viral isolation hampers the establishment of such associations, even though former studies had reported a high prevalence of *Cx. (Mel.) portesi* in endemic areas of ROCV circulation [43,44].

## 5. Conclusions

The detection of arbovirus genome fragments in mosquitoes and antibodies against arboviruses in vertebrates suggest the occurrence of arbovirus transmission cycles in FLONA de Caxiuanã. The results evidence, still, the importance of interactions between wild animals and arthropods hematophagous in the maintenance of these transmissions cycles in FLONA. The results also showed the need of epidemiologic surveillance actions, especially, in the human population resident in FLONA, given their proximity to elements involved in transmission cycles to avoid dispersion of potentially epidemic arboviruses to naive locations due to the frequent movement of people between locations and cities.

## Figures and Tables

**Table 1:** Samples positive for the presence of viral genome from pools of mosquitoes collected at Floresta Nacional de Caxiuanã, in the period of 2014 to 2016.

Pool of Species	Season	Collection Area	Method of Capture	Virus	Homology
*Ur. (Ura.) geometrica*	Dry	ECFPn	CDC (Solo)	MUCV	83%
*Cx. (Mel.) portesi*	Dry	ECFPn	CDC (Solo)	ROCV	92%
*Cx. (Mel.) portesi*	Dry	ECFPn	CDC (Solo)	ILHV	94%
*Cx. (Mel.) portesi*	Dry	ECFPn	CDC (Solo)	BSQV	94%

MUCV: *Mucambo virus*; ROCV: *Rocio virus*; ILHV: *Ilheus virus*; BSQV: *Bussuquara virus*.

## Appendix A

**Table A1:** The antigenic panel used for wild vertebrates selected for the study and tested for Hemagglutination Inhibition (HI) test.

Genus	Virus	Birds	Wild Vertebrates
*Alphavirus*	WEEV	X	X
	EEEV	X	X
	MAYV	X	X
	MUCV	X	X
	AURAV	X	X
	PIXV	X	X
*Flavivirus*	WNV	X	X
	YFV	X	X
	ILHV	X	X
	SLEV	X	X
	ROCV	X	X
	CPCV	X	X
	BSQV	X	X
	NJLV	X	X
*Orthobunyavirus*	MAGV	X	X
	TACV	X	X
	CARV	X	X
	OROV	X	X
	CATV	X	X
	UTIV	-	X
	BLMV	X	-
	APEUV	X	X
	ITAV	X	X
	MARV	X	X
	MURV	X	X
	ORIV	X	X
*Phlebovirus*	URUV	X	X
	BUJV	X	X
	ICOV	-	X

WEEV: *West Equine Encephalitis Virus*; EEEV: *East Equine Encephalitis Virus*; MAYV: *Mayaro Virus*; MUCV: *Mucambo Virus*; AURAV: *Aura Virus*; PIXV: *Pixuna Virus*; WNV: *West Nile Virus*; YFV: *Yellow Fever Virus*; ILHV: *Ilheus Virus*; SLEV: *Saint Louis Encephalitis Virus*; ROCV: *Rocio Virus*; CPCV: *Cacipacore Virus*; BSQV: *Bussuquara Virus*; NJLV: *Naranjal-Like Virus*; MAGV: *Maguari Virus*; TACV: *Tacaiuma Virus*; CARV: *Caraparu Virus*; OROV: *Oropouche Virus*; CATV: *Catu Virus*; UTIV: *Utinga Virus*; BLMV: *Belém Virus*; APEUV: *Apeu Virus*; ITAV: *Itaqui Virus*; MARV: *Marituba Virus*; MURV: *Murutucu Virus*; ORIV: *Oriboca Virus*; URUV: *Urucuri Virus*; BUJV: *Bujaru Virus*; ICOV: *Icoaraci Virus*. X: Virus tested; -: Vírus not tested.

## Appendix B

**Table A2:** Log Neutralization Index (LNI) obtained from NT (Neutralization Test) performed with samples and serological titers to referred viruses, from wild vertebrates captured at Caxiuanã National Forest, between 2014 and 2016.

Genus/Species	Viral Species	Sorological Titer	LNI
*Proechimys sp.*	ILHV	1/320	0.8
*Proechimys sp.*	ILHV	1/640	1.0
*Proechimys sp.*	ILHV	1/640	1.0
*Chelonoidis denticulata*	ILHV	1/160	0.1
*Chelonoidis denticulata*	ILHV	1/160	0.2
*Proechimys sp.*	MUCV	1/640	1.9
*Oecomys sp.*	MUCV	1/320	1.9
*Oecomys sp.*	MUCV	1/40	2.1
*Proechimys sp.*	MUCV	1/40	1.3
*Proechimys sp.*	MUCV	1/160	2.2
*Oecomys sp.*	MUCV	1/80	2.0
*Oecomys sp.*	MUCV	1/80	0.4
*Metachirus nudicaudatus*	MUCV	1/1280	1.9
*Xiphorhynchus spixii*	OROV	1/80	1.8

Footnote: ILHV: *Ilheus Virus*; MUCV: *Mucambo Virus*; OROV: *Oropouche Virus*; LNI: Log neutralization index.
